# Development and validation of a protocol to identify and recruit participants into a large scale study on liver fluke in cattle

**DOI:** 10.1186/s12917-018-1511-3

**Published:** 2018-06-15

**Authors:** Catherine M. McCann, Helen E. Clough, Matthew Baylis, Diana J. L. Williams

**Affiliations:** 1 Department of Infection Biology, Institute of Infection and Global Health, University of Liverpool, Liverpool, UK; 2 Department of Public Health and Policy, Institute of Psychology, Health and Society, University of Liverpool, Liverpool, UK; 3 National Institute of Health Research Health Protection Research Unit in Emerging and Zoonotic Infections, University of Liverpool, Liverpool, UK; 4 Department of Epidemiology and Population Health, Institute of Infection and Global Health, University of Liverpool, Liverpool, UK; 5 Present address: Epidemiology Research Unit, Scotland’s Rural College (SRUC), An Lòchran, Inverness Campus, Inverness, IV2 5NA UK

**Keywords:** *Fasciola hepatica*, Cattle, Farmers, Holdings, Shropshire, Fasciolicide

## Abstract

**Background:**

Liver fluke infection caused by the parasite *Fasciola hepatica* is a major cause of production losses to the cattle industry in the UK. To investigate farm-level risk factors for fluke infection, a randomised method to recruit an appropriate number of herds from a defined geographical area into the study was required. The approach and hurdles that were encountered in designing and implementing this study are described. The county of Shropshire, England, was selected for the study because of the variation between farms in exposure to fluke infection observed in an earlier study.

**Results:**

From a sampling list of 569 holdings in Shropshire randomly drawn from the RADAR cattle population dataset, 396 (69.6%) holdings were successfully contacted by telephone and asked if they would be interested in taking part in the study. Of 296 farmers who agreed to receive information packs by post, 195 (65.9%) agreed to take part in the study. Over the period October 2014 – April 2015 visits were made to 100 dairy and 95 non-dairy herds. During the farm visits 40 faecal samples +/− bulk-tank milk samples were collected and a questionnaire administered. Composite faecal samples were analysed for the presence of *F. hepatica* eggs by sedimentation and bulk tank milk samples were tested with an antibody ELISA for *F. hepatica*. Forty-five (49%) of non-dairy herds were positive for liver fluke infection as determined by the finding of one or more fluke eggs, while 36 (36%) dairy herds had fluke positive faecal samples and 41 (41%) dairy herds were positive for *F. hepatica* antibody. Eighty-seven (45.8%) farmers said that they monitored their cattle for liver fluke infection and 118 (62.1%) reported that they used flukicide drugs in their cattle.

**Conclusions:**

Using a protocol of contacting farmers directly by telephone and subsequently sending information by post, 79% of the target sample size was successfully recruited into the study. A dataset of farm-specific information on possible risk factors for liver fluke infection and corresponding liver-fluke infection status was generated for the development of statistical models to identify risk factors for liver fluke infection at the farm-level.

## Background

Liver fluke infection, caused by the trematode parasite *Fasciola* spp.*,* has an economic impact on livestock production worldwide as a result of both morbidity and mortality. In the UK, where the predominant host species are cattle and sheep, liver fluke infection costs the agricultural industry in the region of £300 million per year due to production losses. Acute clinical disease or sudden death due to liver fluke, a common feature of disease in sheep, is rare in cattle. In dairy cattle liver fluke has been associated with reduced milk production [1, 2], reduced milk fat content and increased calving interval [3]. In high yielding dairy cows, an increase in *F. hepatica* exposure from the 25th to the 75th percentile was associated with a 15% decrease in milk yield [4]. In beef cattle the impact of liver fluke infection on growth rate and weight gain has been less easy to demonstrate [5]. Carcasses from cattle infected with fluke had lower cold weight, lower conformation score and lower fat content than carcasses free of liver fluke in a study of abattoir data in Scotland [6]. Milk yield in dairy cattle or carcass weight of slaughtered beef cattle are reported to decrease on average by 3–5% or 0.5–0.7% respectively [7].

Recent estimates of the prevalence of liver fluke infection in dairy herds in the UK are 80% in 2012 in England, Wales and Scotland [4]; 48 and 86% in 2006–2007 in England and Wales respectively [8]; 72 and 84% in 2005 in England and Wales respectively [9]; and 61–65% in 2011–2013 in Northern Ireland [10]. Traditionally in the UK, most cases of fasciolosis are found in wetter, western areas which provide ideal climatic conditions; however in recent years fasciolosis has emerged in other areas including East Anglia and parts of Scotland [11, 12]. Seasonal risk forecasts that provided an approximation of the potential impact of climate change on fasciolosis in the UK predict an overall long term increase in prevalence of infection in all regions of the UK with an expectation of spatio-temporal variation in risk levels [13].

Liver fluke has an indirect life-cycle involving a snail intermediate host. Principle definitive hosts of *F. hepatica* in the UK are cattle and sheep, however wild herbivorous animals such as deer, rabbits and hares may act as reservoir hosts. Liver fluke have an indirect life cycle involving a snail intermediate host. Undifferentiated fluke eggs are passed out in the faeces of infected animals. The egg hatches to release a miracidium which enters the snail. The main intermediate host is *Galba truncatula*, the amphibious dwarf pond snail. Following further development in the snail, several hundred cercariae are released which then encyst on the pasture. Cattle, sheep and other herbivores become infected when they ingest contaminated herbage, the metacercariae hatch, the newly excysted juveniles burrow through the gut wall and migrate into the liver. [18]. In the presence of suitable definitive hosts, development of *F. hepatica* is dependent primarily on there being suitable environmental conditions for the snail hosts and the intra-molluscan and free-living stages of the parasite. Rainfall and temperature are critical to the development of both the parasite and snail. Other determinants such as soil properties [14], vegetation and altitude [15] and farm management factors [16] may also influence *F. hepatica* transmission. In a recent study of farm management and environmental risk factors for *F. hepatica* exposure in high yielding dairy herds in Great Britain, higher rainfall, grazing boggy pasture, presence of beef cattle on farm, access to a stream or pond and smaller herd size were all associated with an increased rate of exposure [4].

Control of fasciolosis is aimed at reducing the prevalence of disease to permit economic livestock production, as it is unlikely that infection will be eradicated [17]. There are currently no commercial vaccines; hence control is based on the use of anthelminthic drugs to reduce disease and sub-clinical economic losses and the rate of contamination of pastures by reducing fluke egg output [18], together with pasture management, including drainage to reduce the survival of free-living stages and to prevent the establishment of snail populations [19]. There are a number of medicines available to treat cattle for fluke (fasciolicides); these vary in terms of whether they target the immature or adult stages of the parasite and their milk and meat withhold periods. The drug of choice for treatment of fasciolosis is the benzamine derivative triclabendazole, because it is effective against both adult and juvenile fluke [20, 21]; however resistance to triclabendazole has been reported in a number of countries, [21–30] which is a cause for concern especially as triclabendazole is also the drug of choice to treat human fasciolosis. Triclabendazole may only be used at the start of the dry period in dairy animals because of its long withhold period, e.g. milk for human consumption can only be taken from 50 days after treatment with Fasinex® 240 (Elanco Animal Health) [31] In dairy herds with year-round calving, treatment for fluke is likely to be done throughout the year. In the UK, the majority of cattle herds are housed through the winter months; once housed, cattle should no longer be at risk of infection. Hence farmers often treat cattle at a time after housing, to ensure that any fluke present will be killed.

If farmers are able to accurately assess the risk of fasciolosis in their specific location they will be able to make informed decisions on treatment and prevention of disease. Such targeted control programmes should not only result in reduced use of fasciolicides but also lead to better disease control. In the UK, the National Animal Disease Information Service (NADIS) produces a parasite forecast for 10 regions in Great Britain and Ireland (http://www.nadis.org.uk/). This provides farmers with a forecast of the risk of fluke infection on a regular basis, however the coarse spatial resolution of the forecasts do not provide farmers with an accurate assessment of risk of disease to the livestock in their farm.

In an earlier study we developed linear regression models, using a combination of environmental, soil and climatic variables, to describe the observed pattern of exposure to *F. hepatica* in England and Wales using UK postcode area (PCA) to define a spatial unit [32]. The area of these PCAs varied from approximately 150–6000 km^2^, with a mean of 2000 km^2^. Whilst the model explained over 73% of the spatial variation in exposure it did not account for variation of exposure within PCAs. This paper describes the methodology used to recruit nearly 200 dairy and beef enterprises within a small spatial area, to identify more precisely farm-level determinants of *F. hepatica* infection that may explain the variation in prevalence of fluke infection between farms exposed to the same climatic variables. We describe the processes used to identify, contact and recruit farms into the study; once farm proprietors had agreed to participate, visits were made to the farms during which samples were collected to be analysed for evidence of *F. hepatica* infection and a questionnaire administered to collect farm demography, herd health and land and herd management information. Whilst the main aim of this paper is to describe a framework for a truly random recruitment of participants into a study, we also include preliminary data on the prevalence of *F. hepatica* infection in cattle herds in a major farming area of England.

## Methods

### Study population

To estimate the prevalence of exposure to *F. hepatica* in the study area 124 dairy and 124 beef (non-dairy) herds were required for a study with 7.5% precision and 95% level of confidence, based on an assumed herd prevalence of exposure to *F. hepatica* of 76%. Under a data confidentiality agreement with the Animal and Plant Health Agency (APHA), data on all agricultural holdings in England with dairy and/or beef herds were obtained from the Rapid Analysis and Detection of Animal-related Risk (RADAR) cattle population dataset [33]. This database combines the lists held by the Agricultural Census, the Animal Movements Licensing System and the British Cattle Movements Service. Data provided on agricultural holdings included their County Parish Holding (CPH) number, the farm name and address, the Ordnance Survey Easting and Northing and the number of cattle of each breed registered at each holding on 1 May 2014. Most holdings were represented by one farm; however some are used by more than one farm.

Cattle farms in the county of Shropshire in England were chosen as the study population because this population contains large numbers of both dairy and beef farms, and exposure to fluke infection varies from negative to high, based on our 2006/7 survey. A sampling frame of agricultural holdings identified as being located in Shropshire by their postal address, was constructed from the RADAR cattle dataset. The sampling frame comprised 1970 holdings each with between 1 and 2929 cattle registered. Using internet search engines (Google.com and bing.com) phone numbers were found for 1129 holdings; the name of the farm proprietor was also obtained for many of the farms. Two lists comprising 576 holdings with 50 or more non-dairy and 405 holdings with 50 or more dairy breed type cattle were created for a random selection of holdings to be contacted. Based on experience from similar studies, and assuming a participation rate of 40% amongst farmers contacted, a sampling list of 310 beef and 310 dairy herds was required. Random numbers generated in MS Excel were used to select 310 holdings with cattle of each type. Some holdings were selected in both categories; this resulted in a final list of 569 holdings; 300 holdings remained unselected. The list of holdings was randomised to ensure recruitment in a random order to reduce the risk of bias in the time of contacting holdings and subsequently visiting farms and collecting samples.

### Farmer recruitment

Farmers were invited to take part in the study by telephone in the order of the random recruitment list. Between one and seven attempts were made to contact farmers from 15 October 2014–31 March 2015; when attempts had been made to call all the farms the recruiter returned to the beginning of the list. At the beginning of the phone call, the recruiter asked to speak to the proprietor of the farm. Using a prepared script the recruiter then provided information about the study and offered to send further information by post. When possible, eligibility of the farm was determined during the phone call. Inclusion criteria for the study were that there were at least 40 cattle aged at least one year, the cattle had not been treated with an anthelminthic for liver fluke in the previous 12 weeks and the cattle had grazed on pasture on the farm during 2014. The 12-week restriction was not applied to dairy herds with year-round calving which were likely to have year-round liver fluke treatment regimes. If the proprietor was not available and a suitable time to call back could not be arranged, agreement to send the information by post was made. If the farmer was unwilling to receive the information pack, or for any other reason the study information was not sent, the reason was recorded. Herds were classified as dairy or non-dairy (beef suckler, stores/finishers and dairy replacements) based on the details provided during the phone call.

An information pack comprising a Participant Information Sheet, Consent Form and personalised letter was sent to farmers within a week of the recruitment phone call. The Participant Information Sheet provided full details of the study including its objectives, the funding agencies, and what the farmers would be required to do. Farmers were advised that they would be contacted again shortly to be asked if they would be interested in taking part.

One to 3 weeks later, farmers were re-contacted by phone, again using a prepared script, to ask them if they were interested in taking part in the study; if they were, a farm visit was arranged. Again, reasons for not agreeing to a visit were recorded. A maximum of nine attempts were made to re-contact farmers by telephone.

All telephone calls and farm visits were made by the same research scientist. Recruitment was made on a rolling pattern with the aim of obtaining agreement from 10 to 16 farmers per week and to visit 8−10 farms each week. A number of farms located in counties that bordered Shropshire were included in the study – all farms in the RADAR dataset with ‘Shropshire’ in the postal address were included however some were spatially located outside the county border. The study farms were located in an area of approximately 4800 km^2^.

### Description of questionnaire

The questionnaire was piloted on five farms outside the study area. Initially two versions of the questionnaire were used, one for beef and one for dairy farms. After the pilot, a number of adjustments were made including the change to a single version for all herd types.

The final 12 page questionnaire was divided into three sections which covered details about the farm demography and herd health, the herd, pastures and farm management, and cattle production and fertility. Both closed and open-ended questions were used. Questions were designed to determine whether there was a history of liver fluke infection in the herd and to find out if any flukicide drugs were used on the farm and if so, which drugs were used, frequency of treatment and which classes of cattle (and sheep) were treated.

### Farm visit

Signed consent forms were either returned by mail to the study team at the University of Liverpool or collected during the visit.

During the farm visit, which lasted between 1 and 2 h, the questionnaire was completed during a face to face interview with the farmer or their representative. Due to time constraints for six farms the questionnaire was left with the farmer to complete and return by mail to the study team at the University of Liverpool. Three of these six farmers failed to return the questionnaire by post. Interviewees identified areas used for cattle grazing during the 2014 grazing season on farm maps or by providing parcel numbers of the areas grazed. On some farms, photographs were taken of habitat that could potentially harbour *G. truncatula*.

During farm visits, 40 separate faecal samples were collected from individual faecal pats on the pasture or the floor of the shed if cattle were housed. In non-dairy herds, faecal samples were collected from the main cattle group, i.e. from suckler cows, store cattle, fattener cattle or replacement heifers. If the main group of cattle was made up of less than 40 animals, samples were collected from adult cattle, where available. On dairy farms, faecal samples were collected from the cows that were in milk on the day of the visit; additionally, a milk sample was collected from each bulk tank. A preservative bronopolnatamyin (MSI, Nottingham) was added to the milk samples at the time of collection.

### Determination of *F. hepatica* infection status

#### Faecal egg counts

The faecal samples were stored at 4 °C until analysis. For the faecal analysis, the Herdsure® protocol [34] was followed. This uses composite faeces samples to establish the liver fluke infection status of a herd. In brief, 5 g of faeces was taken from each faecal sample and pooled into four 50 g composites for each farm. Each composite sample was mixed with water and a full faecal egg count carried out using the sedimentation technique [35]. By testing four pools of 10 samples there is a 95% confidence level of detecting one positive animal if the within herd prevalence is at least 20%. *F. hepatica* eggs per gram of faeces was calculated for each composite sample. Farms were classified as positive if one or more fluke eggs were detected in at least one of the four composite samples.

#### Bulk tank milk samples

*F. hepatica* antibody levels in the bulk milk tank (BMT) samples were determined using an *F. hepatica* excretory/secretory (ES) antigen specific enzyme-linked immunosorbent assay (ELISA) as previously described [8]. Results were expressed as a percent positivity (PP), which is the optical density (OD) reading of test sample divided by the OD of a positive control, times 100. If the test sample is more strongly positive than the control, the PP will exceed 100%. Herds classed as positive were categorised into low positive (LP) (27 ≤ PP-value < 50); medium positive (MP) (50 ≤ PP-value < 100) and high positive (HP) (PP-value ≥ 100). The sensitivity and specificity of the ELISA to detect herds in which more than 25% of the cows are infected are 96% (95% CI 89–100%) and 80% (95% CI 66–94%), respectively [8].

All farmers were provided with written results for their farms.

### Data analysis

An MS Access database was constructed to hold all data generated relating to farmer recruitment, including telephone call records, for the selected holdings.

The questionnaires were checked manually for inconsistencies and then entered into a database (Epi Info™ version 7.0). Descriptive statistics were estimated and comparisons between the dairy and non-dairy farms performed using STATA (StataCorp. 2015. Stata Statistical Software: Release 14. College Station, TX: StataCorp LP). A Geographical Information System (GIS) (ArcGIS version 10.1) was constructed using data layers of the liver fluke infection prevalence levels at the study farms.

To determine whether there is a statistically significant association between (a) total cattle; (b) grass acreage and farm type (dairy or non-dairy), given that both outcomes are counts the first model of choice is a Poisson log-linear model. The Poisson model assumes that the mean and the variance of the outcome data are the same, however in the case of both outcomes the variance of the data is considerably larger than the mean (mean = 246.9; variance = 29,877.2 for total cattle; mean = 48.1, variance 752.1 for grass acreage). This effect is consistent with most farms having smaller numbers of cattle/acres of grass, and fewer farms having very large numbers of cattle/acres of grass. An ad-hoc way of handling this is to fit the model using quasi-likelihood (a “quasi-poisson model”), which allows the dispersion parameter (which is fixed at 1 in the standard Poisson model) to vary, and to be estimated from the data (in the presence of over-dispersion the estimated dispersion parameter will be greater than 1). The significance of coefficients in a Poisson model which does not allow for over-dispersion will be over-stated, and the effect of allowing for over-dispersion will be to make the estimated coefficient standard errors larger, thereby reducing their significance in the model. We hence fit a quasi-Poisson model to each of the outcomes with farm type (coded 1 = dairy, 0 = non-dairy) as a single covariate.

The Kulldorff spatial scan statistic was used to test whether liver fluke infected farms were randomly distributed within the study area and if not, to identify significant spatial anomalies [36]. Analysis was performed using the Bernoulli model implemented in version 9.4 of the SaTScan software (https://www.satscan.org/). This programme creates circular windows that are moved systematically throughout the geographic space to identify significant anomalies in the spatial distribution of infection. The windows are centred on each of the farms; the maximum window size, to be specified by the user, was defined here as 50% of the farms (i.e. the largest possible cluster would encompass 50% of the farms). For each location and size of the scanning window, SaTScan performs a likelihood ratio test to evaluate whether infection is more prevalent inside than outside that given circular window. Separate analyses were performed for (1) dairy farms with positive BTM samples, (2) dairy farms with positive faecal samples, and (3) non-dairy farms with positive faecal samples. *P* values were determined by Monte Carlo replications of the data set; a 5% significance level was adopted [37].

## Results

### Recruitment

Nine hundred and seventy-four phone calls, lasting a total of 1528 min were made to recruit farmers to the study (Table 1). A minimum of 1 min was recorded for every phone call which was answered, had an answer phone message or was not answered. It was not possible to make telephone calls to 28 farms because the telephone number was not valid. Of the 569 farms selected, contact was made with 396 (69.60%); 296 of those contacted agreed to receive further information about the study. It was not possible to contact 173 (30.40%) farms in the list of selected farms for a variety of reasons (Table 2). Due to the working pattern of many farmers, phone calls were generally made between 9 and 10.30 am, 12–13.30 pm and after 4 pm unless arrangements were made to speak to farmers at other times. Twenty-seven percent of the recruitment telephone calls where a farmer was successfully contacted were made between 5 and 6 pm. Phone calls made between 12 and 1 pm and 4–5 pm each resulted in 12% of the total successful recruitment phone calls.

**Table 1:** Summary of the number of attempted telephone calls made to recruit farms to the study

Number of calls	Number (%) of farms	
	All farms	Farms successfully contacted
0	28 (4.9)^a^	–
1	281 (49.4)	246 (62.1)
2	166 (29.2)	106 (26.8)
3	60 (10.5)	27 (6.8)
4	13 (2.3)	8 (2.0)
5	5 (0.9)	3 (0.8)
6	8 (1.4)	5 (1.3)
7	8 (1.4)	1 (0.3)
Total	569	396

^a^Telephone numbers used were not valid

**Table 2:** Summary of farms which could not be contacted - results of phone calls made

Telephone call result	Number of farms (%)
Number not valid/not connected	28 (16.2)
Wrong number – telephone call answered but not correct for farm	19 (11)
No answer	26 (15)
Answerphone	85 (49.1)
Call answered, unable to speak to proprietor and failed to make contact with farm again	15 (8.7)
Total	173

**Fig. 1:**
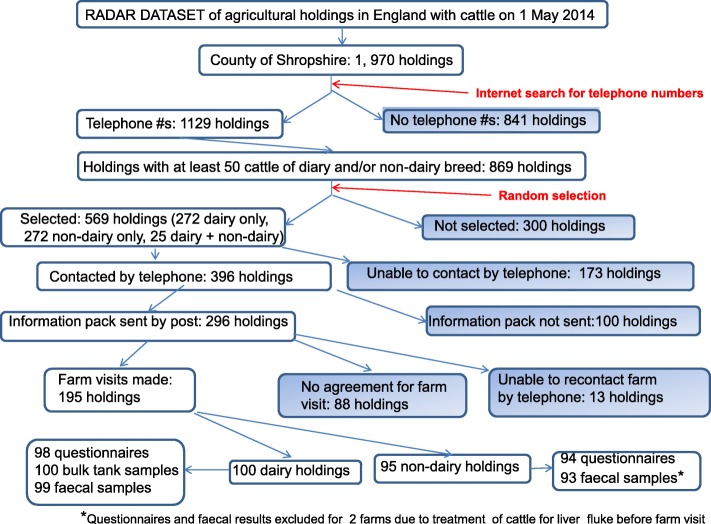
Flow chart showing recruitment of farms into study

Figure 1 provides a flow chart of the recruitment process.

Seven hundred and eighty five follow-up phone calls, lasting 1328 min were made to the 296 farmers who had been sent information packs (Table 3); 195 (65.54%) farmers agreed to take part in the study and a farm visit was arranged. It was not possible to re-contact 13 farmers. The reasons for non-participation in the study are provided in Table 4. Overall 56% of farmers who had been contacted but did not take part were unwilling either because they were too busy or not interested. Other reasons for non–participation included farms not fulfilling inclusion criteria for the study or ill health of the farmer.

**Table 3:** Summary of the number of attempted follow-up phone calls made to farms that had been sent recruitment packs

Number of calls	Number of farms (%)
0^a^	8 (2.7)
1	87 (29.4)
2	73 (24.7)
3	56 (18.9)
4	28 (9.5)
5	19 (6.4)
6	10 (3.4)
7	7 (2.4)
8	4 (1.4)
9	4 (1.4)
Totals	296

^a^The farm visit was arranged at the time of the initial phone call for eight farms

**Table 4:** Reasons provided by farmers for non-participation in study

Reason for non-participation	Recruitment Stage		Total farms (%)
	Initial phone call	Follow-up phone call	
Cattle do not go out	13	2	15 (8)
No cattle/not farming	16	0	16 (8.5)
Not interested or did not want to take part	27	39	66 (35.1)
Too busy/no time	11	29	40 (21.3)
Cattle treated with fasciolicides within the last 90 days	8	8	16 (8.5)
Not enough cattle (< 50)	12	4	16 (8.5)
Retired/ill health/death	11	5	16 (8.5)
Otherwise unsuitable	2	1	3 (1.6)
Totals	100	88	188

Visits were made to 195 farms between 29 October 2014 and 30 April 2015. The farms that participated in the study comprised 100 dairy herds and 95 non-dairy (75 beef suckler, 14 stores/finishers and six dairy replacements). A total of 192 questionnaires were completed. During the visits made to two beef suckler farms it was revealed that the cattle had been treated with fasciolicides within the previous 12 weeks. Faecal samples were collected and analysed for these two farms but the results were excluded from any further analysis. Faecal samples from one dairy and two non-dairy farms were not available for analysis.

### Questionnaires

Farms with a milking herd were classed as dairy farms; all other farms were classed as non-dairy farms. Table 5 provides a summary of the farm size and number of each class of cattle and sheep on each enterprise.

**Table 5:** Summary of farms size (acres) and numbers of cattle and sheep according to class present on the study farms as reported by farmers. Only farms with at least one animal are included for each class

Variable	Dairy				Non-dairy				All farms			
	No. of farms	Mean	Min	Max	No. of farms	Mean	Min	Max	No. of farms	Mean	Min	Max
Total acres	95	346.5	40	5000	91	370.2	27	1600	186^a^	358.1	27	5000
Grassland acres	98	251.1	30	1236	90	221	27	900	188^b^	236.7	27	1236
Dairy cows	98	180.8	45	500	2	21.5	9	34	100	177.6	9	500
Beef cows	3	9	2	17	74	554.6	4	320	77	52.8	2	320
Dairy heifers	88	59.1	5	260	6	162.8	27	280	94	66.7	5	280
Beef heifers	1	4	4	4	61	13.2	1	80	62	13.5	1	80
Calves	90	62.8	2	250	78	41.4	1	310	168	52.9	1	310
Fatteners and stores	32	68.1	5	300	75	91.1	1	468	107	84.2	1	468
Bulls	73	1.8	1	16	64	1.9	1	8	137	1.9	1	16
Total cattle	98	315.4	68	886	92	173.9	25	986	190	246.9	25	986
Sheep	18	246.8	2	2000	64	396.4	3	1900	82	363.6	2	2000
Overwinter sheep	47	323.2	30	1500	25	262.4	50	1000	72	302.1	30	1500

^a^Data not provided by four farms

^b^Data not provided by two farms

The outputs from the quasi-Poisson model are displayed in Table 6. The positive sign of the dairy/non-dairy term in each case suggests that dairy farms have more cattle and more grass acreage than non-dairy farms. The degree of statistical significance of these findings is assessed via analysis of deviance of the fitted models, using an F-test which is appropriate when quasi-likelihood is used. The analysis of deviance confirms that there is a statistically significant positive association between the total number of cattle and the type of farm (F = 38.02, p ≈ 0) but the association between total grass acreage, though positive, is not statistically significant (F = 1.503, *p* = 0.222).

**Table 6:** Output for the quasi-Poisson model to each of the outcomes (a) total cattle and (b) total acreage with farm type (coded 1 = dairy, 0 = non-dairy) as a single covariate

Model	Coefficient	Standard error
Total cattle		
Intercept	5.16	0.08
Dairy/non-dairy	0.59	0.10
Total acreage		
Intercept	5.40	0.08
Dairy/non-dairy	0.13	0.10

### Evidence of liver fluke in herds and use of fasciolicides as reported in questionnaires

Eighty-seven farmers of the 190 farmers who completed the questionnaire (45.8%) said that they monitored their cattle for liver fluke infection. Sixty-one (62.2%) and 26 (28.3%) of dairy and non-dairy farmers respectively said they monitored their cattle for fluke using one or more methods. Table 7 summarises the results reported by farmers of tests carried out to monitor fluke infection on the farms. Some farmers reported that they suspected that liver fluke infection was present but did not have any definitive evidence of fluke infection in their cattle. On 91 (47.9%) farms, farmers reported either recent, past (more than two years ago) or suspected fluke infection (Table 8). Fasciolicide use was reported in cattle and sheep, and in cattle only, on 135 (71.1%) and 118 (62.1%) farms respectively (Table 9).

**Table 7:** Methods used to monitor herds for liver fluke and farmer-reported results of tests used

Method		Dairy herds (*n* = 98)		Non-dairy herds (*n* = 92)		All herds (*n* = 190)	
		Number (%)	Positive (%)	Number (%)	Positive (%)	Number (%)	Positive (%)
*F. hepatica* Bulk tank milk ELISA	Yes	48 (49)	28 (58.3)	NA^a^	–	–	–
	No	50 (51)					
Liver inspection at abattoir	Yes	32 (32.7)	24 (75.0)	23 (25.0)	16 (69.6)	55 (28.9)	40 (72.7)
	No	36 (36.7)	–	14 (15.2)	–	50 (26.3)	–
	Don’t know	2 (2.0)	–	0 (0.0)	–	2 (1.1)	–
	NA	28 (28.6)	–	55 (59.8)	–	83 (43.7)	–
*F. hepatica* faecal egg count analysis	Yes	8 (8.2)	4 (50.0)	6 (6.5)	2 (33.3%)	14 (7.4)	6 (42.86)
	No	90 (91.8)	–	86 (93.5)	–	176 (92.6)	–

^a^*NA* not applicable

**Table 8:** Summary of farmers’ reports of liver fluke infection on their farms

	Dairy herds (*n* = 98)	Non-dairy herds (*n* = 92)	All herds (*n* = 190)
	Number (%)	Number (%)	Number (%)
Evidence of fluke in cattle in last two years	34 (34.7)	19 (20.7)	53 (27.9)
Evidence of fluke in cattle more than two years ago but not in last two years	15 (15.3)	6 (6.5)	21 (11.1)
Suspect fluke (Clinical signs +/− diagnosed in sheep)	8 (8.2)	9 (9.8)	17 (8.9)
No fluke reported on farm	41 (41.8)	58 (63.0)	99 (52.1)

**Table 9:** Summary of reported use of fasciolicide drugs on farms

		Dairy (*n* = 98)	Non-dairy (*n* = 92)	Total (*n* = 190)
		Number (%)	Number (%)	Number (%)
Treat cattle or sheep with fasciolicide within the last two years	Yes	63 (64.3)	72 (78.3)	135 (71.1)
	No	34 (34.7	19 (20.7)	53 (27.9)
	Don’t know	1 (1.0)	1 (1.1)	2 (1.1)
Treat cattle with fasciolicide	Yes	60 (61.2)	58 (63.0)	118 (62.1)
	2 or more years ago	6 (6.1)	10 (10.9)	16 (8.4)
	No	31 (31.6)	23 (25.0)	54 (28.4)
	Don’t know	1 (1.0)	1 (1.1)	2 (1.1)

### Prevalence of *F. hepatica*

**Fig. 2:**
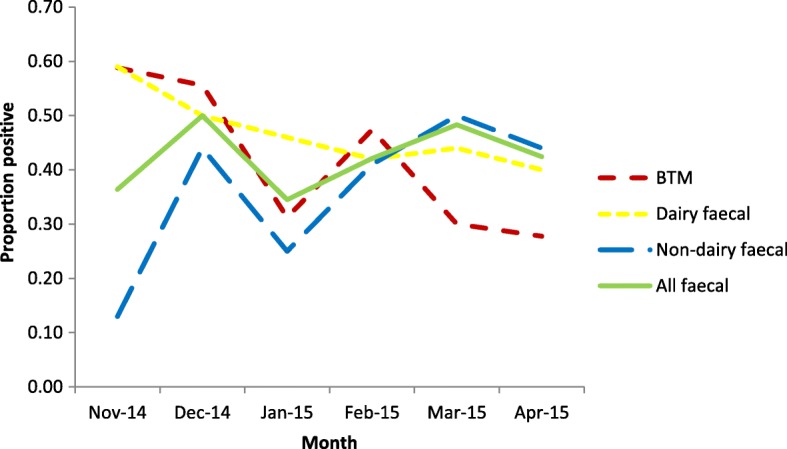
Proportion of farms with positive results in the i. *F. hepatica* sedimentation test for composite faecal samples ii. *Fasciola*-specific bulk tank milk (BTM) ELISA each month (November 2014 – April 2015)

Bulk tank milk samples were analysed for 100 herds and faecal analysis results were available for 190 herds. In the dairy herds 41 (41, 95% CI 31.4–50.6%) of 100 BTM samples tested positive for antibodies to *F. hepatica* (Table 10). Across all herds, 81 (42.6, 95% CI 35.6–49.7%) of 190 composite faecal samples were positive for *F. hepatica* eggs (Table 11). The prevalence of faecal positivity was greater in non-dairy herds compared to dairy herds: 45 (49.5, 95% CI 39.2–59.7%) of 91 and 36 (36.4, 95% CI 26.9–45.8%) of 99 composite faecal samples respectively. Faecal positivity rates each month ranged from 34.5% (January 2015) to 50% (December 2014) of herds tested; bulk tank milk positivity rates each month ranged from 28% (April 2015) to 59% (November 2014) (Fig. 2).

**Table 10:** Results of *F. hepatica* faecal egg count tests according to *F. hepatica* enzyme-linked immunosorbent assay (ELISA) result on dairy farms

BTM ELISA (PP-value)	BTM result category	Faecal result			Total (%)
		Negative	Positive	Not available	
< 27	Negative	45	14	0	59 (59.0)
27–49	Low positive	16	10	1	27 (27.0)
50–99	Medium positive	1	11	0	12 (12.0)
≥ 100	High positive	1	1	0	2 (2.0)
					100

**Table 11:** Results of *F. hepatica* faecal egg count tests in dairy and non-dairy herds

	Farm type					
	Dairy		Non-dairy		All herds	
	n	%	n	%	n	%
Negative	63	63.6	46	50.6	109	57.4
Positive	36	36.4	45	49.5	81	42.6
Total	99	100	91	100	190	100

**Fig. 3:**
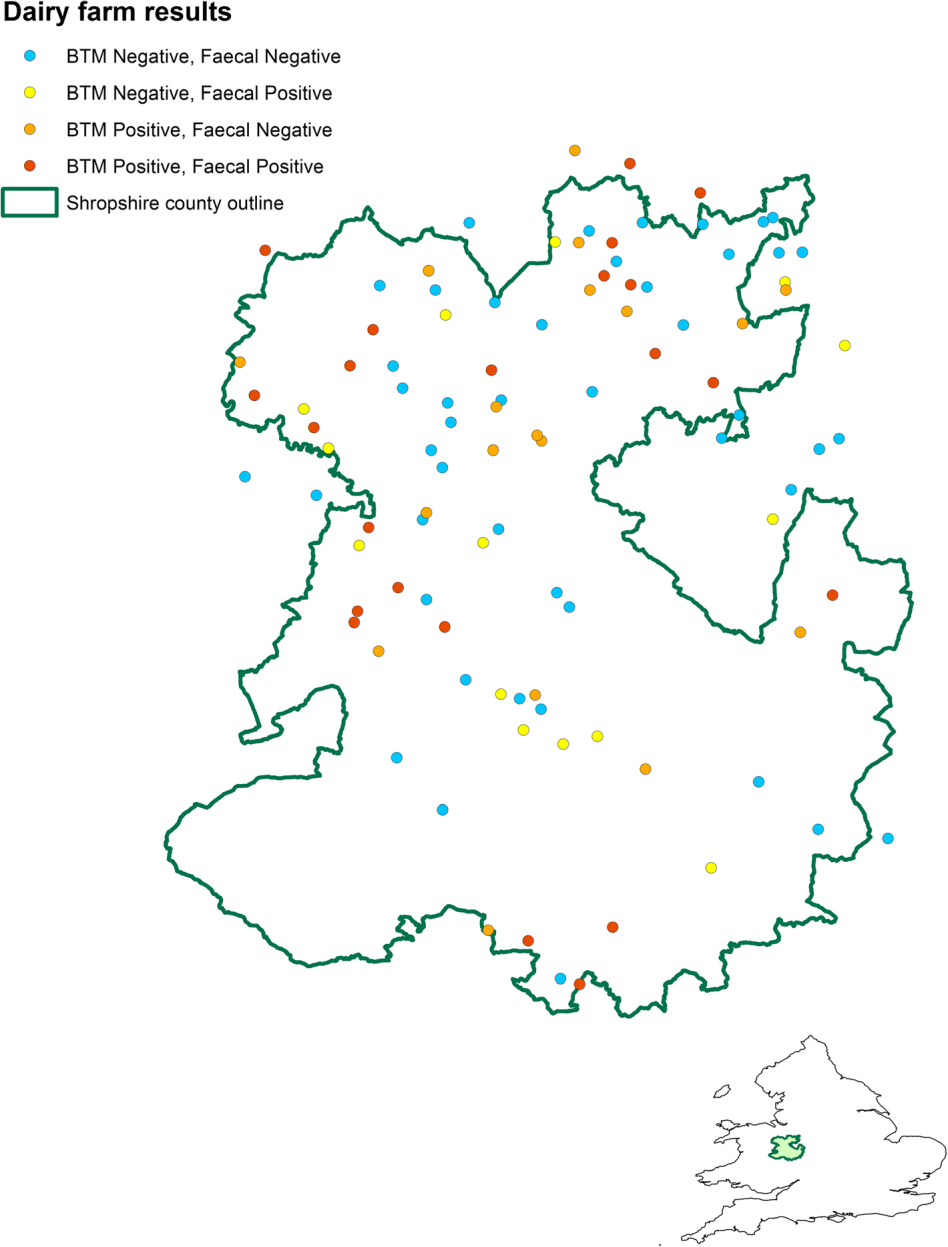
Map showing the location of each dairy farm, georeferenced using X and Y coordinates are jittered randomly within a circular disc of radius 5 km to preserve confidentiality, and colour coded to show liver fluke infection status as determined using a *Fasciola*-specific bulk tank milk (BTM) ELISA and a *F. hepatica* sedimentation test for composite faecal samples

**Fig. 4:**
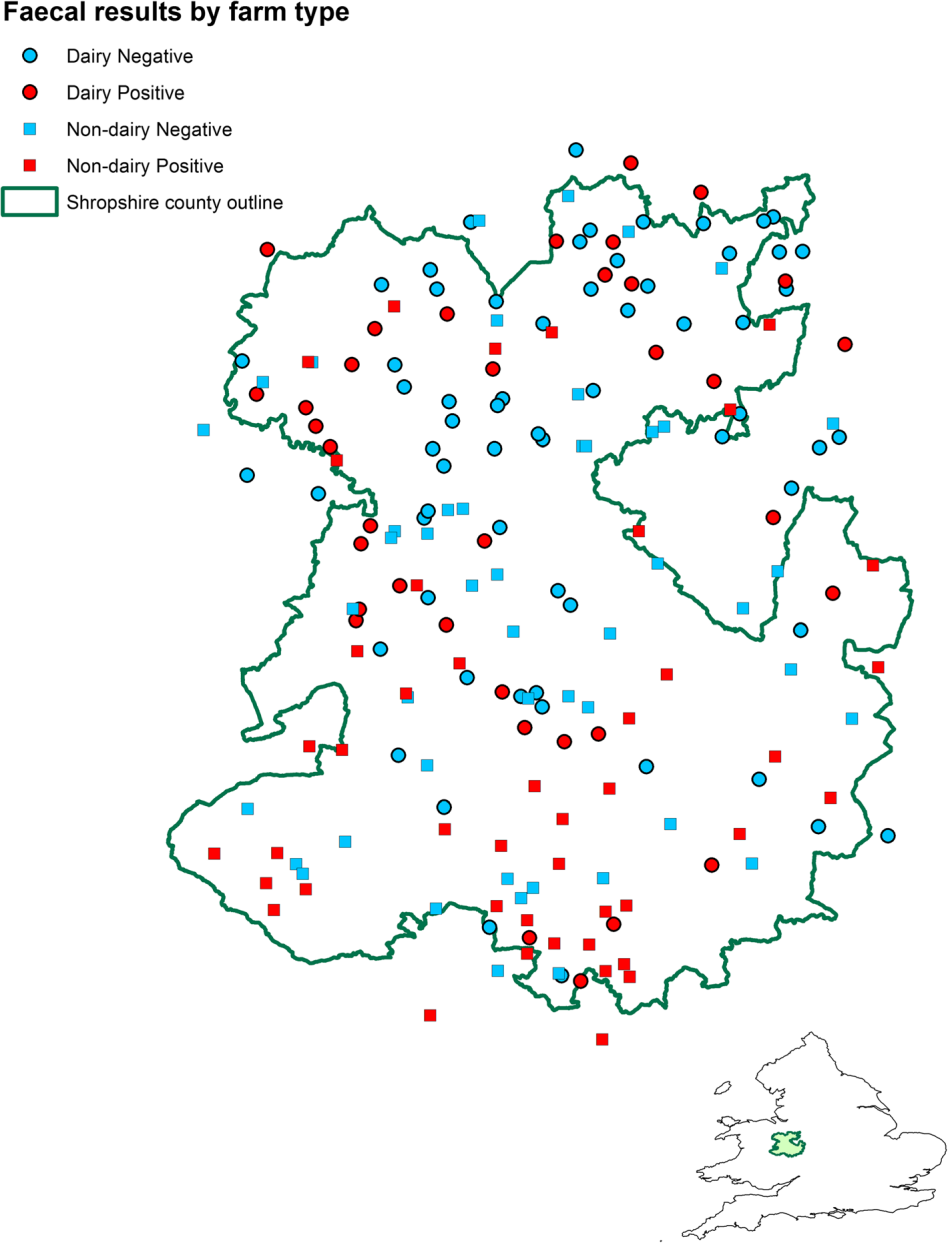
Map showing the location of each farm, georeferenced using X and Y coordinates are jittered randomly within a circular disc of radius 5 km to preserve confidentiality, and colour coded to show farm type and liver fluke infection status as determined using a *F. hepatica* sedimentation test for composite faecal samples

Figures 3 and 4 show the spatial distribution and results for the sampled farms in the county of Shropshire. Figure 3 shows that most of the dairy herds are located in the centre and north of the county. Figure 4, which includes all the sampled herds, demonstrates that *F. hepatica* infection appears to be spread throughout the county with infected and uninfected herds located in close proximity to each other.

### Spatial clustering

Spatial scan statistics revealed no significant anomalies in the spatial distribution of liver fluke infection on dairy or non-dairy farms.

## Discussion

The primary purpose of this paper is to document the methodology used to recruit farms to a study to identify risk factors for liver fluke infection on dairy and beef farms. Many researchers have recruited farmers by contacting them by post with varying results. The RADAR dataset from which we selected our sample provided limited information on the farms; most importantly we did not know whether the farms were still in operation and whether the enterprises were primarily beef or dairy. By contacting everyone by telephone we were able to engage directly with farmers to determine the eligibility of their farms for the study and gauge their interest. Further, by visiting the farms we were able to conduct the questionnaire by face to face interview and provide a diagnostic test to determine the *F. hepatica* status of their herds. Whilst a farmer can collect and send bulk tank milk samples to a laboratory for analysis, the same cannot be said for faecal samples using the exact sampling strategy required for this study.

Only 78.6% of the target sample was successfully recruited. The RADAR dataset from which the names and addresses of cattle farms in Shropshire were obtained did not included contact telephone numbers for the farms, and hence it was necessary to use internet search engines to find the numbers with no guarantee that the telephone numbers were still in service or correct. In addition we were unable to find telephone numbers for 841 of the farms listed for Shropshire in the RADAR list. We found that some of the numbers used to contact farms were incorrect or invalid and also that a number of the farms we contacted were no longer in operation. We have no knowledge of whether the farms for which we were unable to find a telephone number were still in operation. We believe that these issues are likely to be of a random nature and should not result in selection bias. We were unable to contact 30.4% of the farms in the sampling list. With more time, the target sample size could have been achieved by randomly selecting farms from the remaining 300 farms from the list of 869 holdings from which the farms had been selected. This would have required extending the study period; however in the spring time (April 2015) farmers were found to be increasingly occupied with farm activities (e.g. ploughing) and had less time available to accommodate the farm visits despite the longer day length. Also collecting faecal samples was found to be more difficult in the open fields when the cattle were at grass compared to when the cattle were housed so the period of the study was not extended.

Achieving the target recruitment of 124 each of dairy and non-dairy farmers was further complicated because many farms had been included in both the dairy and non-dairy sampling lists. This discrepancy was generally a result of farms of each type often having stores or fatteners of dairy and or beef breeds, for example approximately one-third of the dairy farms kept such animals. Of the farmers who did not participate in the study, 44% were because of inability, rather than unwillingness, to do so. Forty-seven of the farms that did not participate were not eligible to take part by reason of there being no cattle or too few cattle or the cattle did not graze outside. On about 8% of farms contacted the cattle had been treated with a fasciolicide in the last 12 weeks.

Farmers were recruited into this study by telephone. The only information available on the farms recruited into this study was the farm address, location and number and types of cattle as provided in the RADAR dataset. By contacting farms by telephone it was possible to find out whether a farm, listed in the RADAR dataset, was actively operating and whether it fulfilled the criteria to take part in the study. In addition, by contacting farmers by telephone it was possible to obtain information on why farmers who fulfilled the study criteria were unable to participate. This method of recruitment was very time-consuming. Recruitment telephone calls took 25 h, in addition follow-up calls were made, many of which were made outside of normal working hours. The internet search for the telephone numbers took approximately 1 week. However, the personal contact with the farmers resulted in successfully recruiting 195 farms – 65% of the farms that had been sent information packs. Recruitment methods such as contacting farmers in the first instance by mail would have been less time consuming in the first instance, however no information would be obtained on farms from which there had been no response unless letters were returned as undelivered.

This study did offer farmers the incentive of having their herd tested for liver fluke; however it also required farmers to give up 1–2 h of their time to accommodate the visit of the research scientist. It is not possible to determine whether there was any response bias associated with non-participation because the only information available on the non-participating eligible farms was the numbers of cattle as provided by the RADAR dataset. The RADAR database provides a comprehensive repository of data on cattle farms; however the data were often found to be out of date, as a number of farms were no longer operating. Similar issues of RADAR data accuracy have been reported in other studies, for example in a study to determine bluetongue virus vaccine uptake from a random sample of 1866 ruminant holdings, using the RADAR data as a sample frame, 823 questionnaires were returned of which 49 were not valid because they had the wrong address [38].

Other studies on fluke have contacted farms by post, but in all these cases the farmer cohort was under the terms of their milk contract required to participate in research projects funded by the contracting company [4], or used an advisory service [39, 40] hence these were not truly randomised studies. Four hundred and 50 dairy farmers were invited to participate in a survey on liver fluke in Ireland and were sent a questionnaire and a bulk tank milk sampling kit [39]. Contacted farmers either participated in Teagasc (Irish Agriculture and Food Development Authority) discussion groups or were selected by Teagasc dairy advisors, which is a probable explanation for the high response rate of 82%. In a postal survey of Irish dairy farms to obtain information on parasite control practices, 96% of the 312 farmers who were sent the questionnaire responded. The study farms were randomly selected from the Irish Cattle Breeding Federation database and all were members of HerdPlus®, a management decision support tool for dairy farmers [40]. Participation in the study was entirely voluntary and non-incentivised. In a observational study on liver fluke of high-yielding dairy farms who were contracted to supply milk to a major-supermarket chain, 58% of 606 farmers who were sent a questionnaire by post either completed a paper or online version [4]. Reasons for non-completion of the questionnaire were not obtained, however all the farms contacted were operating as the researchers obtained bulk tank milk samples for all of them from National Milk Laboratories. In these three studies, enough information was known about the farms to believe that they were operating and that recruitment by post would result in successful contact with the farms concerned.

In this study, 189 questionnaires were completed during the farm visit; however only three of the six farmers who were asked to return the questionnaire by mail returned them. This may be due to a lack of interest in the study, but is unlikely to introduce bias because of the small number of farms involved.

In this study, by choosing to contact farms by telephone using landline telephone numbers that were obtained using internet search engines, farms were excluded from participating if they could not be contacted using the number found. Some of these farms may have no longer been in operation. However, it is also possible that, with the increasing popularity of mobile phones, some farmers may no longer use landline telephones. Of 64% of the farms in the sampling list that could not be contacted, the telephone was either not answered or there was an answerphone; however failure to contact farms was unlikely to be a cause of non-response bias. In addition, randomising the order in which farms were visited should have prevented any sampling bias in the results according to type, size and management system of the farms.

Fluke eggs were found in nearly 50% of the non-dairy herds. Only 28% of the non-dairy farmers reported that they monitored their cattle for fluke infection with 37% of these reporting that they either had evidence of fluke in their herds or suspected that their cattle were infected. However 63% of non-dairy farmers reported using fasciolicide; it would appear that many farmers use drugs without knowing if their animals are infected.

In dairy herds where 62% of farmers reported that they monitored their herds for fluke infection by one or more method, fluke eggs were found in 36% of herds and 41% of herds were positive by the BTM *F. hepatica* ELISA. The reported use of fasciolicides in 61% of dairy herds was similar to that reported in non-dairy herds although the timing of treatment was different.

The majority of dairy herds sampled had year round calving and in 50% of herds, cows were treated at the beginning of the dry period which may not coincide with when they are most at risk of infection. The BTM antibody ELISA detects liver fluke antibodies that are present in milk and is a good screening test for lactating dairy cows that contribute their milk to the BTM sample. Antibodies may persist 4–10 weeks after treatment; hence the ELISA cannot distinguish between current and recent exposure [41]. Discordant results were found between the *F. hepatica* BTM antibody ELISA and the faecal analyses in 32 herds. Eighteen herds were positive by the BTM ELISA but negative for fluke eggs. There are several possible explanations for this result. Firstly whilst the specificity of the faecal egg count test is high, the sensitivity is low, 30–70% depending on method and study area [7]. In this study we used a composite test which adds a layer of complexity. Also, in herds with year-round calving and routine dry cow treatment for fluke, there is likely to be a continuous entry of newly calved uninfected cows that may still be antibody positive, thus resulting in a positive BTM ELISA and negative faecal egg count. Also *F. hepatica* antibodies are produced in the serum 2–4 weeks after infection hence an ELISA that detects antibody in the milk can detect early, pre patent infection whilst a faecal egg count will only detect patent infection.

The finding of 14 dairy herds which were negative by the BTM and positive for faecal eggs may be explained by the characteristics of the BTM ELISA used in the study. A positive BTM ELISA result identifies herds in which least 25% of cows have been exposed which means that some of the cows from herds with a within-herd prevalence of less than 25% may be infected. The method of collection of faecal samples for the study was random, hence samples from infected cows was probable in some herds which had low levels of infection. The composite method for faecal samples is designed to detect a positive herd with 95% certainty whilst the BMT ELISA is designed to detect herds with more than 25% of cattle infected.

In this study the prevalence of fluke infection was 55% in dairy herds by BTM ELISA and/or a composite faecal egg count test, while the reported use of fasciolicide drugs was over 60%. Whilst previous data are not available for the herds in this study, other researchers have found that in endemic regions of Spain, Switzerland and Belgium, where repeated surveys were carried out, there was little change in prevalence of infection despite regular fasciolicide treatment [7].

The prevalence of fluke as determined by the composite faecal egg count test in the non-dairy herds was 49%. The detection of fluke eggs in faeces requires a patent infection – egg laying commences 10–12 weeks after ingestion of metacercariae. In the UK fasciolosis is a seasonal disease, development of the parasite in the intermediate host occurs between May and October if the weather conditions are appropriate, and cattle acquire infection in the autumn. In this study, farms were recruited and visited over a seven month period from October 2014 – April 2015. It is likely that some of the cattle which were sampled in October or November may have become infected with metacercariae in the autumn and may have tested negative for liver fluke eggs because the infection was not patent at the time of sample collection.

In this study we did not find any spatial pattern in the spread of negative and positive results, this is important because the farms were situated in a small area and would have had similar climates.

## Conclusions

In this study we successfully recruited 79% of the target sample of farms into a study of liver fluke in cattle. Whilst recruitment by telephone was time consuming, by engaging directly with farmers we were able to determine why farmers were able/not able to participate. The information collected from each farm, together with environmental data, is being used to develop models to identify risk factors associated with different levels of infection at the farm level.

## Abbreviations

APHA: Animal and Plant Health Agency
BMT: Bulk milk tank
CI: Confidence interval
CPH: County Parish Holding
ELISA: Enzyme-linked immunosorbent assay
ES: Excretory/secretory
GIS: Geographical Information System
HP: High positive
LP: Low positive
MP: Medium positive
NADIS: National Animal Disease Information Service
OD: Optical density
PCA: Postcode area
PP: Percent positivity
RADAR: Rapid Analysis and Detection of Animal-related Risk
Teagasc: Irish Agriculture and Food Development Authority
